# Mitochondrial transfer balances cell redox, energy and metabolic homeostasis in the osteoarthritic chondrocyte preserving cartilage integrity

**DOI:** 10.7150/thno.96723

**Published:** 2024-10-07

**Authors:** Angela C. Court, Ana María Vega-Letter, Eliseo Parra-Crisóstomo, Francesca Velarde, Cynthia García, Alexander Ortloff, Rolando Vernal, Carolina Pradenas, Patricia Luz-Crawford, Maroun Khoury, Fernando E. Figueroa

**Affiliations:** 1 Cell for Cells, Santiago, Chile.; 2 Laboratory of Nano-Regenerative Medicine, Faculty of Medicine, Universidad de los Andes, Santiago, Chile.; 3 Laboratory Cell and Molecular Immunology, CIIB, Faculty of Medicine, Universidad de los Andes, Santiago, Chile.; 4 Departamento de Ciencias Veterinarias y Salud Pública, Facultad de Recursos Naturales, Universidad Católica de Temuco, Temuco, Chile.; 5 Facultad de Odontología, Universidad de Chile, Santiago, Chile.; 6 Consorcio Regenero, Chilean Consortium for Regenerative Medicine, Santiago, Chile.; 7 IMPACT, Center of Interventional Medicine for Precision and Advanced Cellular Therapy, Santiago, Chile.

**Keywords:** cartilage regeneration, chondrocytes, mesenchymal stromal cells, mitochondrial transfer, osteoarthritis

## Abstract

Osteoarthrosis (OA) is a leading cause of disability and early mortality, with no disease modifying treatment. Mitochondrial (MT) dysfunction and changes in energy metabolism, leading to oxidative stress and apoptosis, are main drivers of disease. In reaction to stress, mesenchymal stromal/stem cells (MSCs) donate their MT to damaged tissues.

**Methods:** To evaluate the capacity of clinically validated MSCs to spontaneously transfer their MT to human OA chondrocytes (OA-Ch), primary cultured Ch isolated from the articular cartilage of OA patients were co-cultured with MT-labeled MSCs. MT transfer (MitoT) was evidenced by flow cytometry and confocal microscopy of MitoTracker-stained and YFP-tagged MT protein. MT persistence and metabolic analysis on target cells were assessed by direct transfer of MSC-derived MT to OA-Chs (Mitoception), through SNP-qPCR analysis, ATP measurements and Seahorse technology. The effects of MitoT on MT dynamics, oxidative stress and cell viability were gauged by western blot of fusion/fission proteins, confocal image analysis, ROS levels, Annexin V/7AAD and TUNEL assays. Intra-articular injection of MSC-derived MT was tested in a collagenase-induced murine model of OA.

**Results:** Dose-dependent cell-to-cell MitoT from MSCs to cultured OA-Chs was detected starting at 4 hours of co-culture, with increasing MT-fluorescence levels at higher MSC:Ch ratios. PCR analysis confirmed the presence of exogenous MSC-MT within MitoT^+^ OA-Chs up to 9 days post Mitoception. MitoT from MSCs to OA-Ch restores energetic status, with a higher ATP production and metabolic OXPHOS/Glycolisis ratio. Significant changes in the expression of MT network regulators, increased MFN2 and decreased p-DRP1, reveal that MitoT promotes MT fusion restoring the MT dynamics in the OA-Ch. Additionally, MitoT increases SOD2 transcripts, protein, and activity levels, and reduces ROS levels, confering resistance to oxidative stress and enhancing resistance to apoptosis. Intra-articular injection of MSC-derived MT improves histologic scores and bone density of the affected joints in the OA mouse model, demonstrating a protective effect of MT transplantation on cartilage degradation.

**Conclusion:** The Mitochondria transfer of MSC-derived MT induced reversal of the metabolic dysfunction by restoring the energetic status and mitochondrial dynamics in the OA chondrocyte, while conferring resistance to oxidative stress and apoptosis. Intra-articular injection of MT improved the disease in collagenase-induced OA mouse model. The restoration of the cellular homeostasis and the preclinical benefit of the intra-articular MT treatment offer a new approach for the treatment of OA.

## Introduction

Osteoarthritis (OA) is a chronic condition of high prevalence and a main health burden contributing to global disability 1. It is also an independent risk factor for mortality 2. Options for disease modifying treatments are lacking. Disease progression is driven by mechanical and proinflammatory stress, aging, and metabolic factors responsible for cartilage damage 3. Indeed, chondrocytes are the only cell type within mature cartilage 4, and several studies point to a mitochondrial bioenergetic failure of chondrocytes 5,6 characterized by profound ATP depletion associated with disease 7. This leads to oxidative stress, further mitochondrial DNA damage, and increased rates of apoptosis and cartilage matrix degradation 8,9. Recently, mitochondrial dynamics have been described as the main component that governs this process 10, such that increased mitochondrial fission leads to mitophagy and energetic failure, while fusion improves mitochondrial function and energy production, inhibiting chondrocyte apoptosis 11.

Due to the chondrogenic, anti-inflammatory, and regenerative properties of Mesenchymal stromal cells (MSCs) 12,13, their therapeutic use in OA has been widely tested in Clinical trials by our group and others 14,15,16, and animal disease models 17,18. Recent research points to an alternative approach, given that the transfer of mitochondria (MT) from donor MSCs to damaged tissues 19 conveys potent effects on target cells 18-21. Under metabolic stress, intercellular MT transfer can restore bioenergetics and maintain the viability of recipient cells, including chondrocytes 23.

There are presently no disease modifying treatments for OA, a disease with great social and clinical impact. The recent description of the tissue and cell regenerating properties of the transfer of MSC-derived mitochondria (MitoT) open a “window of opportunity” to intervene the OA process 24.

Given the central role of MT in the energetic failure of OA, we sought to assess 1) if human umbilical cord-derived MSCs (UC-MSCs) would transfer their MT to human chondrocytes and 2) if MitoT could restore some of the dysfunctional traits of the OA chondrocyte, including metabolic activity, ATP production, mitochondrial dynamics and oxidative stress management. Since we found evidence for both phenomena, we proceeded then to demonstrate that the intra-articular injection of UC-MSC led to the improvement of disease in an animal model of OA. These findings might represent a new therapeutic strategy in the treatment of OA.

## Materials and Methods

***Human OA cartilage samples and chondrocyte isolation:*** Articular cartilage samples were obtained from osteoarthritic (OA) hip or knee joint replacement surgery with prior informed consent. Patients fulfilled the American College of Rheumatology (ACR) classification criteria for OA 25. Articular cartilage was aseptically removed from the subchondral bone and cut into small pieces into Pronase solution (2.5 mg/ml of Pronase, Sigma-Aldrich, St. Louis, MO, USA) prepared in Dulbecco's Modified Eagle's Medium (DMEM, Corning Inc, NY, USA). Fragments were then incubated overnight with Collagenase type II (250 U/ml, Sigma-Aldrich) supplemented with 1X antibiotics (Penicillin 200 units/mL, Streptomycin 200 μg/mL and Amphotericin B 5 µg/mL) from Invitrogen (Waltham, MA, USA), at 37ºC under constant agitation. Cells were washed, filtered, and seeded (20.000 cells/cm^2^) with fresh culture media with 10% Fetal Bovine Serum (FBS, Lonza Group AG, Basel, Switzerland), 1 mM L-glutamine and 1% Penicillin/Streptomycin (P/S) (Life Technologies, Carlsbad, CA, USA) for primary human chondrocyte (Ch) cultures. All subjects gave their informed consent for inclusion before they participated in the study. All procedures were approved by the Ethics Committee of Universidad de los Andes, Chile (#CEC202033) and in conformity with the Helsinki Declaration.

***MSCs isolation and culture:*** Umbilical cord (UC) derived MSCs were isolated from healthy donor umbilical cords and characterized according to the International Society for Cell and Gene Therapy (ISCT) criteria, under GMP condition, as we have previously described 26. All experiments were performed using early passage (P3-P7) UC-MSCs cultured in the culture media described above. All cells were grown in humidified incubation chambers at 37 °C with 5% CO_2_.

***MitoT detection by co-culture assay and flow cytometry analysis (FACS):*** Different MSC:Chondro ratios were used for co-culture assays, using UC-MSCs labeled with MitoTracker Green (MTG, Molecular Probes, Eugene, OR, USA), and primary human chondrocytes previously stained with CellTrace Violet (CTV) dye (Thermo Fisher Scientific, Waltham, MA, USA), following the manufacturer's recommendations. For the 1:1 ratio, an equal number of each cell type (50.000 cells) were plated in 12-well plates. After co-culture, cells were collected, washed with PBS and labeled with the Near-IR fluorescent reactive dye for cell viability (Live/Dead Fixable Near-IR Dead Stain Kit, from Thermo Fisher Scientific). Data were acquired using FACS Canto II, analyzed with the FlowJo v.10 software and expressed with the mean fluorescence intensity (MFI) values.

***Confocal Microscopy:*** Confocal microscopy of chondrocytes was performed with a Leica TCS SP8 confocal laser system using MitoTracker Green (MTG), MitoTracker Deep-Red (MTDR) or MitoTracker Orange (MTO) for staining MSCs MT, and either MTG or CellTrace Violet (CTV) dye for chondrocyte labeling, according to manufacturer's instruction. When indicated, cells were stained with Hoechst (nuclear staining) (Invitrogen, H3570), after 24-48 hours-post mitoception. For TNT visualization in co-cultured Chs, we used UC-MSCs transfected with a mitochondrial yellow fluorescent protein plasmid (mito-YFP) donated generously by Jorge Toledo, Redeca Director (Universidad de Chile).

***ATP determination:*** According to the manufacturer's instructions, ATP production was measured using the CellTiter-Glo® Luminescent Cell Viability Assay (Promega, Madiso, WI, USA). For co-culture experiments, ATP levels were measured from 3.000 FACS-sorted MitoT^+^ or MitoT^-^ primary human OA chondrocytes, previously cultured with UC-MSC for 6, 12 or 24 hours.

For artificial MT transfer, OA Ch was seeded in an opaque 96-well plate (3.000 cells/well) and mitocepted in a ratio 1:1. Oligomycin A (Sigma-Aldrich, #75351) was added at 1 ug/mL as control of ATP productive active-MT. For each sample, 50 µL of CellTiter-Glo® Reagent was added and the plate was incubated for 30 min at room temperature, followed by a luminescence reading using a BioTek FLx800 microplate reader.

***Mitochondrial mass and copy number analysis by PCR:*** To follow exogenous MSC-MT over time, total DNA was isolated from mitocepted primary chondrocytes, collected at different time points after mitoception with MSC-MT (1-9 days), using QIAamp DNA Mini Kit (Qiagen, Hilden, Germany) or Cells and Tissue DNA Isolation Kit (Norgen Biotek Corp, Thorold, ON, Canada), following the manufacturer's instructions. Mitochondrial DNA (mtDNA) was quantified by amplification of a part of the cytochrome c oxidase mitochondrial gene: MT-CO1 forward primer 5'GGCCTGACTGGCATTGTATT-3' and reverse primer 5'TGGCGTAGGTTTGGTCTAGG-3'. Nuclear DNA was quantified using the following primers: 18S nuDNA forward primer 5'-TAGAGGGACAAGTGGCGTTC-3' and reverse primer 5'-CGCTGAGCCAGTCAGTGT-3', targeting the nuclear 18S rRNA gene. Mitochondrial mass was calculated as mtDNA/nuDNA ratio. Mitochondrial DNA Copy Number was calculated based on the equation: a number of copies = (amount of DNA (ng) x 6.022e23) / (length of amplicon (bp) x 1e9 x 650 (average mass of 1bp of dsDNA)), using a standard curve created by qPCR amplifying serial dilutions of the mt-CO1 amplicon (178bp, previously purified), and 30 ng of sample's DNA. qPCR was performed in duplicate on a Stratagene Mx3000P with the use of Brilliant II SYBR Green Master Mix (Agilent Technologies, Santa Clara, CA, USA).

***Mitochondria isolation and artificial transfer (Mitoception):*** UC-MSC MT were isolated from previously MitoTracker labeled donor cells using the Mitochondria Isolation Kit (Thermo Fisher Scientific), following manufacturer's instructions. MT isolated from 1 to 10x10^6^ MSCs and resuspended in MSC culture media were maintained on ice until artificial transfer (within 2-3 hours). Mitoception was performed on primary Chs seeded the day before (100.000 chondrocytes per well on a 12-well plate), using the amount of MSC MT corresponding to MSC:Ch ratios of 1:10, 1:5 and 1:1. The following day cells were washed with PBS 1X and used for experimental procedures.

***Single Nucleotide Polymorphism (SNP) analysis:*** For PCR and targeted Sanger sequencing analysis, part of the mtDNA domain within the D-loop was amplified using the high-fidelity KOD Hot Start DNA Polymerase kit according to the manufacturer's instructions (Novagen, Sigma-Aldrich). Predicted size was verified by DNA gel electrophoresis and Sanger sequencing was performed at Universidad Católica de Chile. The sequence chromatograms of the mtDNA from the MSCs, analyzed using 4Peaks (www.nucleobytes.com), revealed several single nucleotide polymorphisms (SNPs) for creating unique mutational sites; among which T16153C and G228A were chosen to distinguish between the exogenous MSC-MT from the MT derived from the parental chondrocytes. Using the amplification refractory mutation system (ARMS) principle 27, for each SNP two sets of primers were designed to generate unique PCR products for MSC-donated or chondrocyte endogenous mitochondrial transcripts: one pair detecting the WT transcript, and one pair detecting the SNP transcript. Also, the forward (FW) outer and reverse (Rev) outer primers flanking the editing site were used as a PCR positive control to ensure that the editing region was detectable. Relative expression levels were calculated using the 2-ΔCt method.

***Determination of Mitochondrial Membrane Potential (ΔΨm), TMRM assay:*** After 24 hours post mitoception, 100.000 MitoT^+^ or non mitocepted OA Ch cells were stained with 50 nM tetramethylrhodamine (TMRM) (Invitrogen), for 20 minutes at 37ºC. The depolarization potential was tested by treating the cells with 100 µM CCCP (carbonyl cyanide 3-chlorophenylhydrazone (CCCP, Sigma-Aldrich) for 5 minutes before data acquisition using FACS Canto II Flow Cytometer. Histograms showing PE fluorescence were analyzed and the mean fluorescence intensity (MFI) of each sample was calculated using FlowJo v.10 software.

***Metabolic measurements by extracellular flux analysis:*** To assess the metabolic impact of MitoT on OA-Chs, the SeaHorse XF96 Flux Analyzer instrument (SeaHorse Bioscience, North Billerica, MA, USA) was used, to measure oxygen consumption rates (OCR) and the extracellular acidification rate (ECAR) in real time in mitocepted chondrocytes, in order to test mitochondrial respiration and glycolysis, respectively. Primary OA-Chs were mitocepted with MSCs-MT at MSC: Chondro ratios of 1:1, as previously described, and cells were plated on a 96-well plate (samples run in quadruplicates) with XF buffer. OCR (pmol/min/cell) was measured for 80 min using the following mitochondrial inhibitors: Oligomycin (10 µM), FCCP (2.5 µM), Antimycin A (20 µM) and Rotenone (20 µM). ECAR (mpH/min/cell) measurements were quantified using Glucose (10 mM) and Oligomycin (10 µM). All SeaHorse measures were normalized to the number of cells counted in each well seeded before SeaHorse experiments.

***Western Blot analysis:*** Primary human chondrocyte cells were collected after 24, 48 or 72 hours post-mitoception with MSCs-MT and resuspended with 50 µl of cold RIPA buffer containing protease/phosphatase inhibitors (Cell Signaling Technology, Danvers, MA, USA). Samples were then sonicated for 5 min at high intensity and centrifuged at 13200 rpm for 15 min at 4°C to discard cellular debris. Supernatants containing proteins were collected and protein quantification was performed using the Bradford method following the manufacturer's instructions (Bio-Rad, Hercules, CA, USA). 20-30 µg of total protein were loaded and run in 10% SDS-PAGE gels. 5% of BSA in PBS-Tween 0,01% was used for blocking and primary antibodies were used in a dilution of 1:1000 prepared in PBS-Tween 0,01%, overnight with agitation at 4°C (all antibodies from Cell Signaling Technology: SOD2 #13141, TOM20 #42406, α-OPA1 #67589, α-MFN1 #14739, α-MFN2 #9482, α-DRP1 #14647, α-phospho-DRP1 Ser616 #4494 and α-β-Actin #3700). Secondary fluorescent antibodies were prepared in a 1:20000 dilution of 5% BSA in PBS-Tween (0,01%) (secondary antibodies from Invitrogen: α-mouse Alexa Fluor 680 #A32729 and α-rabbit Alexa Fluor 800 #A32735). Finally, fluorescence was detected in the Odyssey®CLx and analyzed with Image Studio Lite software (version 5.2).

***Mitochondrial network status analysis by fluorescent microscope:*** Human OA chondrocytes were mitocepted with UC-MSCs MT at a ratio of 1:1. Chondrocytes at 24 or 48 hours post-mitoception were stained with MitoTracker Orange (as described above), fixed with 4% of PFA for 15 min at room temperature and stained with nuclear Hoechst in dark. Finally, cells were washed, and coverslips were mounted on slides with mounting media. Images were obtained in a Leica TCS SP8 confocal microscope. For each experimental group at least 10 images were selected from two independent experiments. Images were analyzed using an ImageJ macro tool (mito-morphology) to quantify the number of MT per cell, perimeter, and area.

***Collagenase-induced mouse model*** ***of Osteoarthritis (CIOA):*** C57BL6 mice from The Jackson Laboratory (Bar Harbor, ME, USA) were kept at a pathogen-free animal facility of Universidad de los Andes with water and food *ad libitum*, according to ARRIVE guidelines for animal care and research, with protocols approved by the Institutional Animal Care Board and by the Ethics Committee of Universidad de los Andes, Santiago, Chile (CEC nºCEC202033). Ten to 12-week-old male mice were used for OA induction, injecting 1U/5μL of Clostridium histolyticum type VII collagenase solution (Sigma-Aldrich #C2399) into the left knee joint, at days 0 and 2 of the experiment. For the therapeutic effect of MitoT, CIOA mice were randomized and injected with 200.000 MSC or isolated MT derived from the same amount of cells, on days 7 and 14 of the experiment. The control (non OA) Sham group were contralateral legs injected with sodium chloride. Mice were euthanized at day 14 or 42, and knee joints were harvested for Micro-CT imaging and histological analysis. A pre-established exclusion criteria was used in case of animal suffering, following the recommendations of the American Veterinary Medical Association (AVMA) or with a clinical score greater than 4 (scale 0 to 5).

***Isolation of mouse chondrocytes from articular cartilage for human MT detection:*** Ten to 12-week-old C57BL6 male mice were intra-articularly injected with isolated MT derived from 200.000 UC-MSCs into the left knee joint. The next day, mice were euthanized and the articular cartilage surfaces were collected in PBS containing 10X antibiotics (penicillin and streptomycin). Then, cartilage fragments were incubated for 1 hour at 37ºC in 1mg/ml Pronase solution (Sigma-Aldrich #10165921001), followed by a 6 hour incubation with 1mg/ml Liberase^TM^ (Sigma-Aldrich # 5401127001). Chondrocytes were liberated by pipetting, filtered, washed and seeded in complete culture medium. Mouse chondrocytes were collected after 5-6 days in culture for qPCR and confocal microscopy analysis. RNA was isolated using RNeasy Micro Kit from Norgen and following the manufacturer's recommendations, and cDNA was prepared in a reverse-transcription reaction using the SuperScript II Reverse Transcriptase (Thermo Fisher Scientific). Specific human B2M, human MT and mouse b-actin primers were used, and their sequences are available as previously reported.

***X-ray microtomography (Micro-CT) analysis:*** The knee joint samples were analyzed through X-ray microtomography, Micro-CT, using the SkyScan 1278 Micro-CT equipment provided by Universidad de Chile, under characteristics defined by the equipment operator. Three-dimensional reconstruction was performed using NRecon software. For Micro-CT images analysis, the DataViewer program was used to select the respective volume of interest (VOI) to be analyzed for each sample. The VOI generated was analyzed using the CTan program, which allows obtaining a 2D (bone mineral density, grayscale index, among others) and 3D analysis of the respective area of the sample by repeatedly selecting a section of the joint/bone. For all the samples, the analysis was performed in 4 zones: lateral subchondral, medial subchondral, lateral femur, and medial femur.

***Histopathological analysis:*** Mice knee joint samples were collected in 4% PFA solution and then transferred to 5% formic acid, where they were kept for one week. The cartilage staining process 28, was performed by soaking the slices in 0.1% Safranin O (Sigma: S8884) solution for 10-15 minutes (for proteoglycans and glycosaminoglycans staining), followed by washing with Milli Q water; the slices were immersed in 0.1% Fast Green (Sigma: F7258) solution for 10 minutes (allowing bone/collagen staining), washed again, then proceeding to the quantification of histopathological score of each evaluated slice (analysis of bone and articular cartilage degradation). The analysis of bone and articular cartilage degradation (OARSI score) was done, in a blinded fashion, in 5 sections per slice of the knee based on what was observed in a nanozoomer (scanner equipment that digitizes histological samples into high-resolution images). Specific features were used in order to provide a joint damage score, determined as a "grade" that corresponded to the progression of OA depth in the cartilage, which is used for the calculation of the OA Score in conjunction with the stage, a term used to describe the horizontal extent of cartilage below that grade. The maximum score can reach a value of 30, where the higher the value the greater the OA damage. Both the grade and stage scales were standardized by the laboratory itself based on the evaluation scale defined by Pritzker 29.

***Masson's Trichome staining:*** Mice knee joints samples were fixed in 10% neutral buffered Formalin and decalcified with EDTA for 4-5 days. Paraffin blocks were prepared and thin sections (3µm) were cut. To evaluate the intensity of collagen staining, sections were submitted to a Masson's trichrome staining (Sigma-Aldrich, #HT15) to visualize collagen in blue and staining procedure was performed according to the manufacturer's instructions. Two images, at 40x of magnification, were taken using a light microscope (Leica DM500, Wetzlar, Germany) connected to a digital camera (Leica ICCW50). Staining intensity was quantified by transforming the blue channel, obtained with color deconvolution tool (ImageJ software 1.8.0), in Trichrome images to gray scale, and then selecting the cartilage area and bone area to measure the mean gray scale values to the tissue for the selected area, obtaining a normalized relationship between bone and cartilage with the formula (mean cartilage intensity/mean bone intensity). Finally, the mean of the images was obtained.

***Cell viability by Annexin V / PI assay:*** For cell viability and apoptosis assays, primary human OA chondrocytes were mitocepted with MSCs-MT at MSC: Chondro ratios of 1:1 or 2:1, as previously described. Apoptosis was induced with increasing concentrations of hydrogen peroxide (H_2_O_2_) incubated for 24 hours, or with a 3-hour treatment with 50 µM of Menadione 30,31 (stress inductor, #M5625, Sigma-Aldrich). Then, cells were washed with PBS and labeled with annexin V - APC (1:50) (Biolegend, #640919) and propidium iodide (PI) (1:50) (Biolegend, #421301) with a commercial binding buffer for 20 min at room temperature and protected from light. In the final step, dyes were diluted by adding 100 µL of binding buffer, and cells were analyzed by flow cytometry using FACS Canto II and analyzed with the FlowJo v.10 software.

***Mitochondrial and general ROS measurements:*** MSCs-MT or No MitoT human chondrocyte cells were collected 24 hours post-mitoception with MSC-MT at MSC: Chondro 1:1 ratio, and incubated with 25 µM of Menadione for 20 min at 37°C. After which, cells were collected and stained with 2.5µM of MitoSox (mitochondrial superoxide indicator, Invitrogen, #M36008) or CM-H2DCFDA (general oxidative stress indicator, Invitrogen, #C6827) for 30 min at 37°C. Cells were collected and analyzed immediately using FACS Canto II, and the FlowJo v.10 software.

***RNA extraction and quantitative RT-PCR:*** For qRT-PCR total RNA was isolated from 200.000 MitoT^+^ or non mitocepted primary human OA chondrocytes, after 48 hours post-mitoception with MSC-MT (1:1 ratio), previously resuspended in RL lysing buffer (Norgen,17200), using RNeasy Micro Kit from Norgen and following the manufacturer's recommendations. Complementary DNA was prepared in a reverse-transcription reaction using 100 ng of RNA and SuperScript II Reverse Transcriptase (Thermo Fisher Scientific). 5 ng of template mRNA and 5 µM of each forward and reverse human specific SOD2 primers were used: forward primer 5'-gggagatgttacagcccaga-3' and reverse primer 5'-agtcacgtttgatggcttcc-3'. Data were expressed as relative gene expression or fold mRNA expression using the 2-ΔCt method and normalized to 18S rRNA housekeeping gene.

***Superoxide Dismutase (SOD) activity:*** Human OA-Chs were mitocepted with UC-MSCs MT at a 2:1 ratio, as previously described. After 24 hours, cells were collected (400.000-600.000), washed with cold PBS 1X and sonicated for 5 min. After 10 min centrifugation (1500xg at 4ºC) the supernatant was used immediately to measure SOD2 activity, using the Superoxide Dismutase (SOD) Colorimetric Activity Kit (Invitrogen, #EIASODC), following the manufacturer's recommendations. Standard curve and samples were measured using a Tecan infinite M200 Pro microplate reader, at 450nm.

***TUNEL assay:*** Apoptotic cell death for cultured cells was analyzed using the TUNEL assay kit (Abcam, #ab66110) per manufacturer's instructions. Briefly, MitoT^+^ OA-Chs and No MitoT control chondrocytes, previously seeded on 12 mm round coverslips, were incubated for 15 minutes at 37°C, with 25 µM Menadione. Cells were then fixed with 4% paraformaldehyde, labeled with BrdU-Red and DNA labeling solution for 60 min at 37ºC, mounted and analyzed using SP8 Leica confocal microscope. Apoptotic cell death in cartilage from mice knee joints samples was detected using the TUNEL assay kit (Fluorescence, 488nm; Cell Signaling #25879), according to manufacturer's instruction. Paraffin-embedded histological sections were deparaffinized, rehydrated, and incubated with EnVisionTM Flex Target Retrieval Solution for antigen retrieval, and apoptotic chondrocyte cells were visualized using SP8 Leica confocal microscope. For quantitative analysis, number of TUNEL+cells were counted (10x magnification) in the cartilage region (ROI, 10x magnification), and expressed per unit area.

***Statistical Analysis:*** *In vitro* tests were performed using different biological replicates in at least three independent experiments. Differences were assessed by unpaired t-test as indicated for each figure legends, and considered statistically significant for p values of < 0.05. *In vivo* results were analyzed with non parametric paired group statistics. The p values generated with non-parametric analysis (Mann-Whitney *U* test) for two groups were considered statistically significant at p < 0.05 (*), p < 0.01 (**), or p < 0.001 (***). Analyses were performed with GraphPad Prism 9.0.1 software (GraphPad Software, San Diego, California, USA), and presented as mean ± SEM.

## Results

### Mitochondrial transfer (MitoT) from UC-MSCs to chondrocytes *in vitro*

**Figure 1:**
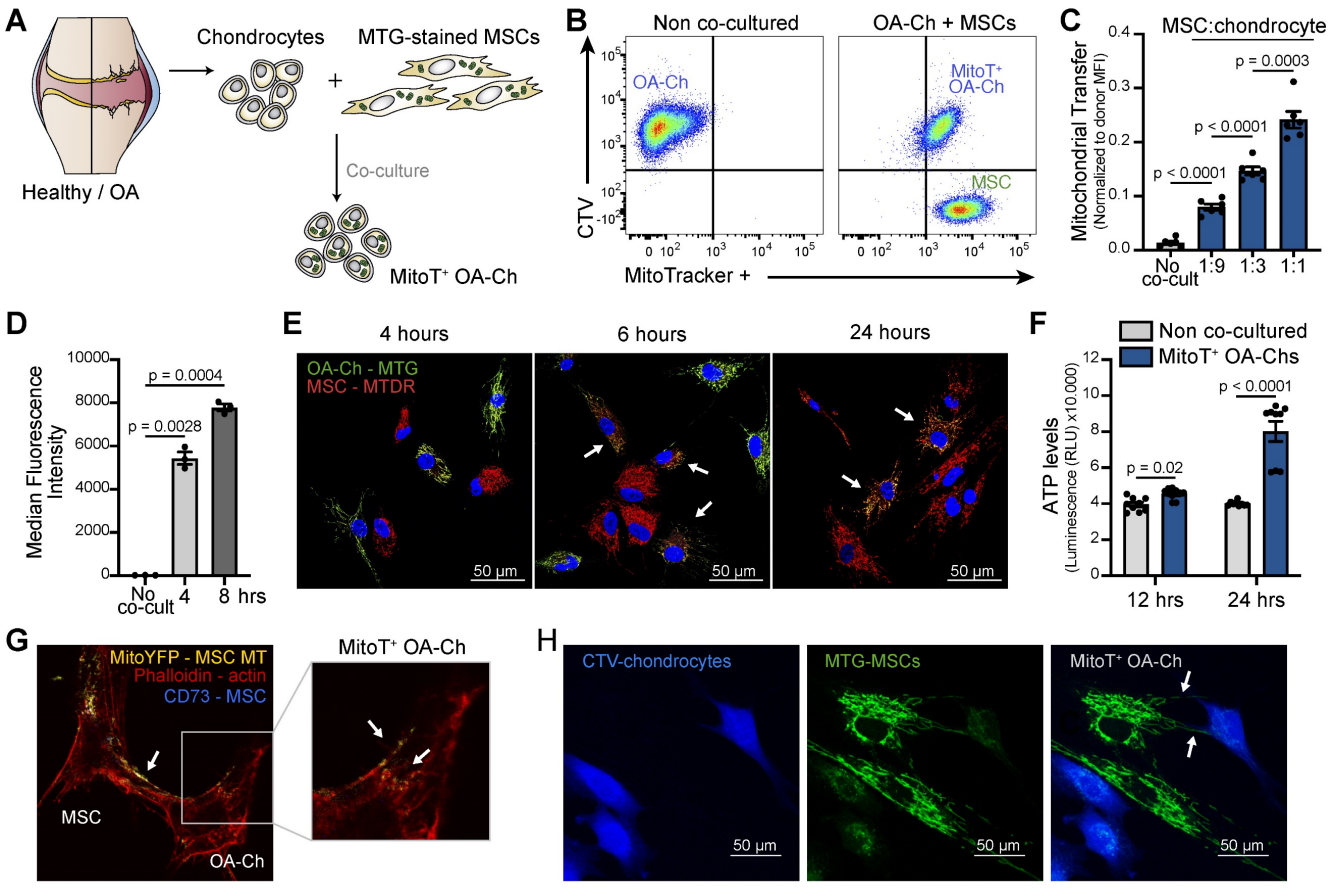
** MT Transfer from UC-MSC to human OA chondrocytes. (A)** Experimental design of MT transfer (MitoT) from UC-MSCs to human chondrocytes isolated from OA patients (OA-Chs) for co-culture experiments.** (B)** Representative FACS plots of MitoT to human OA-Chs stained with CTV and co-cultured with MTG-labeled human MSCs in a 1:1 ratio (right panel). Control OA-Chs with no MSC co-culture (left panel). **(C)** Normalized MFI by FACS analysis of MitoT^+^ OA-Chs after 24 hr of co-culture at increasing MSC:chondrocyte ratios (*n* = 6 patient samples).** (D)** FACS analysis of MitoT to OA-Chs at early co-culture times (4 and 8 hr) with 1:1 UC-MSC:OA-Ch ratio (*n* = 3 patient samples). **(E)** Confocal microscopy of MitoTracker-DeepRed (MTDR) stained MSCs co-cultured with MTG-labeled OA-Chs at different time points. White arrows point to MitoT^+^ within OA-Chs, showing both endogenous and exogenous MT. **(F)** ATP level of MitoT^+^ FACS-sorted OA-Chs after 12 or 24 hour co-culture with UC-MSCs, compared to no MSC co-culture control (non co-cultured OA-Chs) (*n* = 3 patient samples).** (G)** Confocal microscopy images of MitoT seen within tunneling nanotubes (TNT-like) bridging MitoYFP-transfected donor UC-MSCs to OA-Ch, after 24 hrs of co-culture. **(H)** Confocal microscopy imaging of human CTV-stained OA-Chs at 24 hrs of co-culture with MTG labeled UC-MSCs. Tunneling nanotube-like structures (white arrows) are seen between donor and recipient cells (scale bar: 50 µm). Graphs show mean ± SEM and statistical analysis by Student's t-test. All replicates are biological.

To assess the role of MSC-derived MT in the restoration of the mitochondrial dysfunction that characterizes OA cartilage, we first evaluated the capacity of human UC-MSCs to spontaneously transfer their MT, with no external stimulation, to isolated human OA chondrocytes (OA-Chs). Primary cultured Chs isolated from the articular cartilage of OA patients were co-cultured with UC-MSCs previously labeled with an MT fluorescent probe, MitoTracker Green (MTG) (Figure 1A). Both flow cytometry (FACS) analysis and confocal immunofluorescence showed cell-to-cell MitoT as evidenced by the detection of donor MSC-MT within the OA-Chs (MitoT^+^) (Figure 1B and Figure S1A). MitoT exhibited a dose-response effect when donor MSCs were co-cultured at increasing MSC:Ch ratios (3.2-fold increase comparing 1:1 to 1:9 ratio) (Figure 1C and Figure S1B). Cell-to-cell transfer occurs within 4 hours of co-culture (p = 0.0028) (Figure 1D and Figure S1C). Also, a double color labeling of chondrocyte-MTs with MTG and MSC-derived MTs with MitoTracker Deep Red (MTDR) disclosed the presence of both endogenous and exogenous MT in the target OA-Chs after short co-culture times (Figure 1E).

### Metabolic effects of the cell-to-cell MitoT from MSCs to OA-Chs

Given that OA-Chs exhibit a decreased number of MT per cell and reduced cellular ATP levels 7 we explored the metabolic effects of cell-to-cell MitoT from MSCs to OA-Chs. Significant increases of ATP in FACS-sorted MitoT^+^ OA-Chs were found after 12, and 24 hours of co-culture, compared to non co-cultured Chs (2-fold increase at 24 hours) (Figure 1F). These changes paralleled the increases triggered by MitoT in mitochondrial mass and copy number in MT recipient OA-Chs (Figures S1D-E).

To confirm the occurrence of MitoT from UC-MSCs to OA-Chs, we also tested mito-YFP transfected MSC-donor cells, evidencing the same phenomenon by confocal immunofluorescence (Figure 1G). These images pointed to tunneling nanotube (TNT) like structures containing labeled-MT in our co-cultured experiments (Figures 1G-H).

### Metabolic rescue after artificial MitoT on OA-Chs

**Figure 2:**
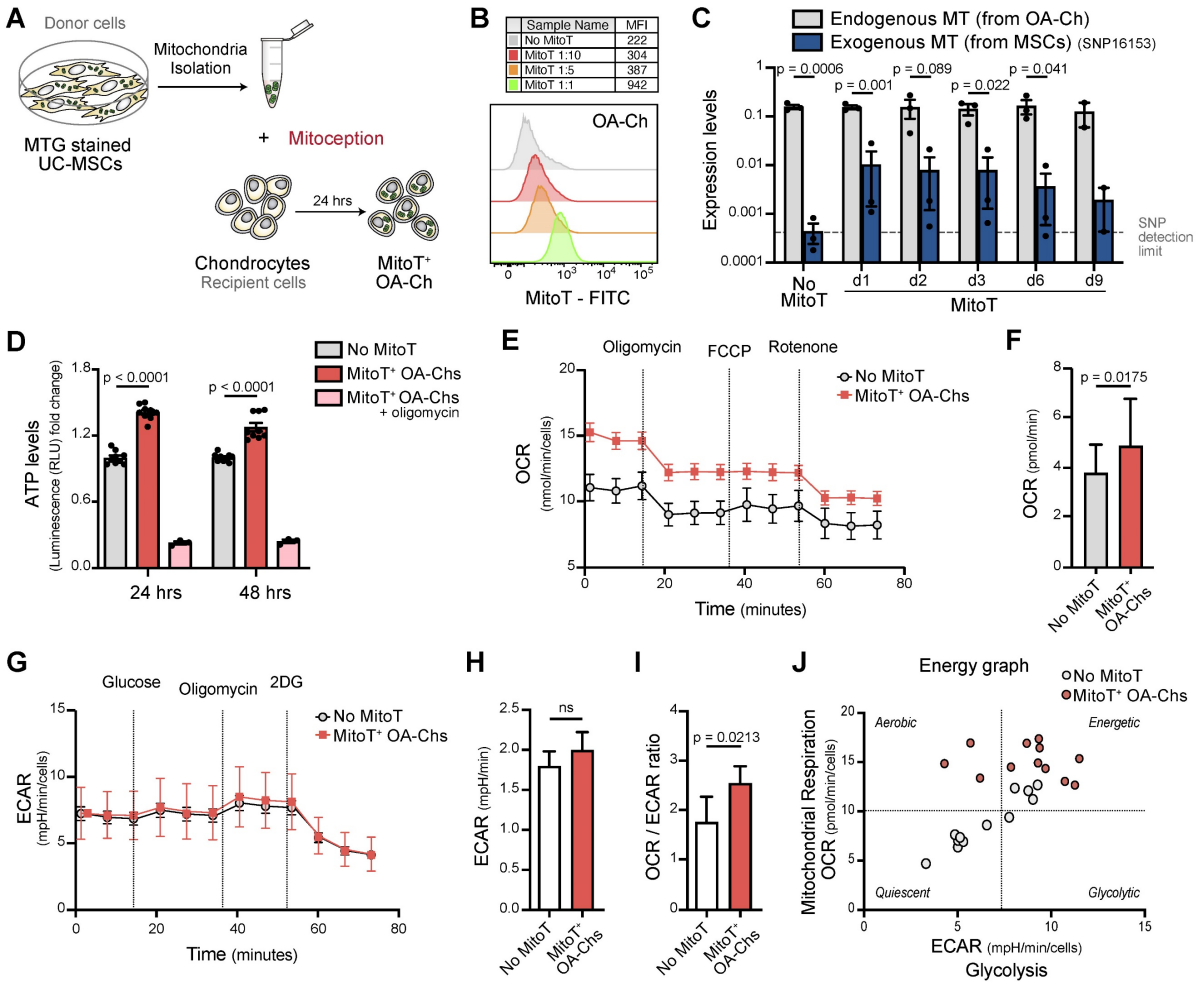
** Artificial MT Transfer to human OA chondrocytes. (A)** Diagram for the artificial MT transfer (mitoception protocol) of human OA chondrocytes (OA-Chs).** (B)** Representative FACS histograms of MitoT^+^ OA-Chs transferred with increasing amounts of UC-MSC-derived MT (MitoT), isolated from the equivalent number of MSCs according to previously tested cell ratios. Non mitocepted control in gray histogram (No MitoT).** (C)** Persistence of MSC-derived MT in OA-Chs (days 1 through 9) according to SNP-PCR analysis of human-specific MSC mitochondrial SNP (16153 T-to-C) gene expression levels, in OA-Chs collected at different time points after mitoception with MSC-MT doses equivalent to a 1:1 cell ratio, compared to non mitocepted chondrocytes (No MitoT) (*n* = 3 patient samples).** (D)** Relative ATP levels of MitoT^+^ OA-Chs, at 24 and 48 hours post-mitoception, compared to non-mitocepted control chondrocytes (No MitoT). Light blue bars depict mitocepted cells treated with 1ug/mL of oligomycin as control of ATP productive active-MT (*n* = 3 patient samples). **(E-F)** Oxygen Consumption Rate (OCR) analysis measured in an XF96 analyzer (Seahorse) extracellular flux analyzer of MitoT^+^ OA-Chs after 24 hrs post-mitoception with UC-MSC derived-MT in doses equivalent to a 1:1 cell ratio, compared to non mitocepted control chondrocytes (No MitoT) (*n* = 4 patient samples).** (G-H)** Extracellular Acidification Rate (ECAR) analysis measured in in an XF96 analyzer (Seahorse) extracellular flux analyzer of MitoT^+^ OA-Chs after 24 hrs post-mitoception with UC-MSC derived-MT in doses equivalent to a 1:1 cell ratio, compared to non mitocepted control chondrocytes (No MitoT) (*n* = 4 patient samples).** (I)** Oxphos/glycolysis ratio on MitoT^+^ OA-Chs after 24 hrs post-mitoception with UC-MSC derived-MT compared to No MitoT control (*n* = 4 patient samples). **(J)** Energetic plot representing the metabolic status of mitocepted-OA-chondrocytes compared to non-mitocepted OA-chondrocytes. Graphs show mean ± SEM and statistical analysis by Student's t-test. All replicates are biological.

Given the therapeutic potential of MSC derived MT, we assessed the effect of the direct transfer of isolated MT, functional and actively producing ATP 20 32, to the OA Ch (Figure 2A).

Dose-dependent uptake of these MSC-derived MT, assessed by FACS analysis, was evidenced employing MT in quantities equivalent to MSC:Chondro increasing ratios (Figure 2B and Figure S2A).

The persistence of the exogenous MSC-MT within OA-Chs over time was evaluated by quantifying the increase in mitochondrial mass and MFI of donor MT post-MitoT. Both qPCR of mitochondrial gene expression and MFI levels of MSC-derived MT within OA-Chs were increased in MitoT^+^ OA-Chs compared to non mitocepted cells (Figures S2B-C). Exogenous MSC-derived MT could be detected in target OA-Chs up to 9 days post-MitoT. This was according to a Single Nucleotide Polymorphisms (SNP) PCR analysis (Figure 2C) with specific primers for MSC mitochondrial SNP expression levels within MitoT^+^ sorted OA-Chs (Figures S2D-F).

To evidence that metabolic changes in MitoT^+^ OA-Chs following co-culture experiments were related to MT function, we checked for ATP levels, MT membrane potential (MMP), and cell-metabolic flux analysis of mitocepted OA-Chs. These results showed that artificial MitoT was associated with a significative increase (p < 0.0001) in ATP levels in MitoT^+^ OA-Chs compared to control Chs (Figure 2D). Although MitoT did not increase the MMP of the OA-Ch as we expected, MitoT^+^ OA-Chs still exhibited a greater depolarization of the MMP (decreased MFI levels) in response to the blockage of mitochondrial oxidative phosphorylation with CCCP (uncoupling agent treatment) when compared to non mitocepted cells. We infer that MitoT conveys an increase in the depolarizing response as assessed by the depolarization capacity (Δψm) (Figures S3A-B). This led us to further assess the metabolic impact of MitoT on OA-Chs, with extracellular flux analyzer, Seahorse technology, measuring oxygen consumption rates (OCR) and the extracellular acidification rate (ECAR) in real time, in mitocepted Chs. We observed a significant increase (p = 0.0175) in the OCR rates (an indicator of OXPHOS activity) in MitoT^+^ OA-Chs compared to non MitoT cells (Figures 2E-F), with no change in ECAR rates (indicative of the glycolytic activity of the cells) (Figures 2G-H). Thus, the OXPHOS/Glycolysis ratio was increased (p = 0.0213) in MitoT^+^ OA-Chs compared with controls (Figure 2I) pointing to a metabolic switch towards an activated energetic state of the OA chondrocyte in response to MitoT (Figure 2J). Taken together, these results suggest that MSC derived MitoT tends to restore the metabolic activity of the dysfunctional OA-Ch.

### MitoT promotes MT fusion, restoring MT dynamics in the OA chondrocyte

**Figure 3:**
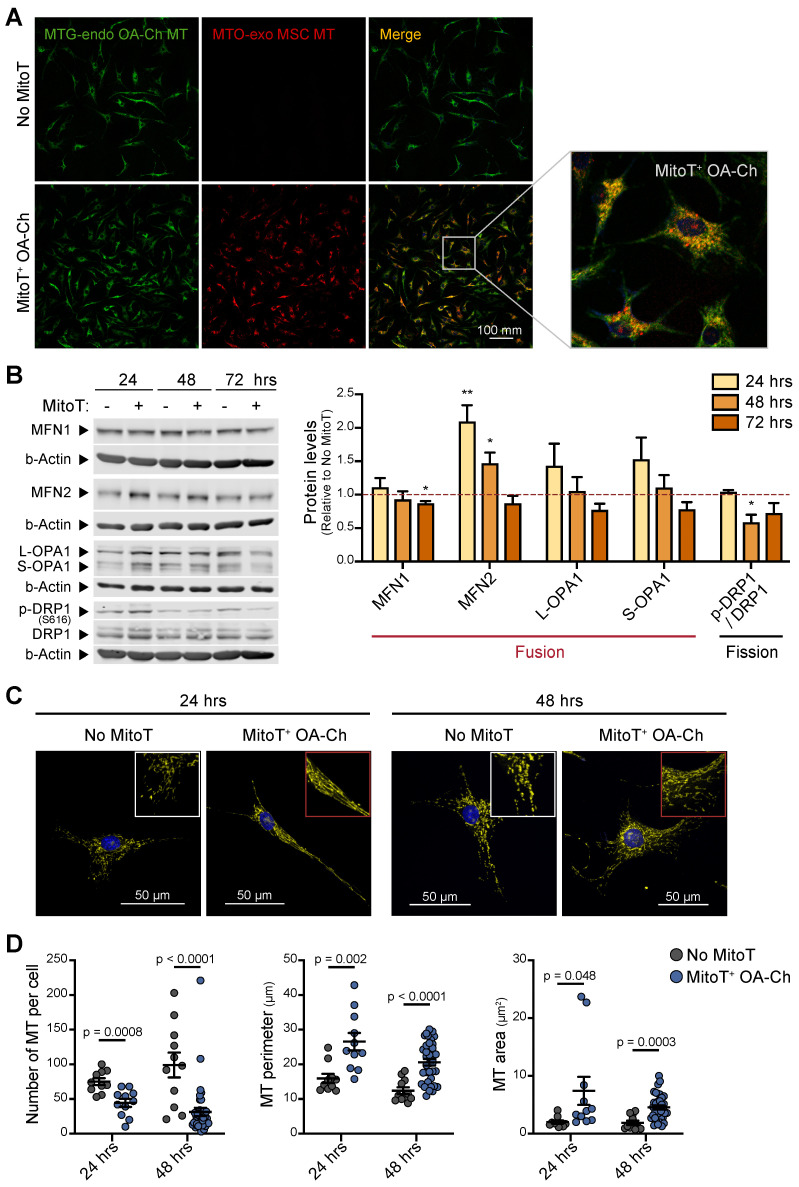
** MitoT promotes mitochondrial fusion in OA chondrocytes. (A)** Representative confocal microscopy of MitoT^+^ OA-Chs, after 24 hours post mitoception with MitoTracker-Orange (MTO)-labeled MSC-MT (bottom row), compared to non-mitocepted chondrocytes (No MitoT) stained with MitoTracker-Green (upper row). The white box represented the zoom of merged images. **(B)** Western Blot analysis of proteins related to the mitochondrial fusion/fission process in MSC-MT mitocepted OA-Ch. Representative blots at 24, 48 and 72 hours post mitoception (left panel) and average bar graphs (right panel) from three different OA patient samples. The graph depicts fold expression of tested proteins in MitoT^+^ OA cells relative to No MitoT cells, which are set at 1. **(C)** Representative confocal microscopy of OA-Chs stained with MTO and Hoechst (nuclear blue stain) at 24 and 48 hours post mitoception with MSC-MT, showing a more elongated mitochondrial network (middle column) compared to a fragmented MT network in non-mitocepted cells (left column). **(D)** Quantitative analysis of mitochondrial morphology in MitoT^+^ OA-Chs after mitoception with MSC-MT, compared to no-mitocepted controls (No MitoT), assessed by confocal microscopy as mentioned above (*n* = 10-38 independent cells analyzed per group). Graphs show mean ± SEM and statistical analysis by Student's t-test (** p < 0.01; * p < 0.05). All replicates are biological.

Mitochondrial dynamics has been reported to direct the energy balance of the OA-Chs 11. Thus, we assessed the status of the MT network in response to MitoT. Confocal microscopy imaging with double color staining of OA-Ch MT and donated MT, was indicative of the fusion of donor cell-derived MT with the native OA-Ch-MT (Figure 3A). Generally, MT fusion is associated with an increased respiration rate and improved coupling efficiency 33. Quantitation of the main fusion/fission regulator proteins also confirmed that MitoT promotes a fusion state of the mitochondrial network, since we detected an increase in Mitofusin-2 (MFN2) at 24 (p < 0.01) and 48 hours after MSC-derived MitoT, and a decrease (p < 0.05) in phosphorylated Ser616-DRP1 (p-DRP1) /DRP1 ratio -that promotes MT fission, compared to non-MitoT OA-Chs (Figure 3B). Confocal image analysis also evidenced this fused MT status in response to MitoT, revealing a more extensive and elongated mitochondrial network in terms of MT perimeter and MT area in the MitoT^+^ OA-Chs, at 24- and 48-hours post mitoception (Figures 3C-D). These results indicate that MitoT promotes MT fusion, restoring one of the main aspects of the unbalanced mitochondrial dynamics reported in the OA-Chs.

### Therapeutic effect of intra-articular injection of UC-MSC derived MT in a murine model of OA

**Figure 4:**
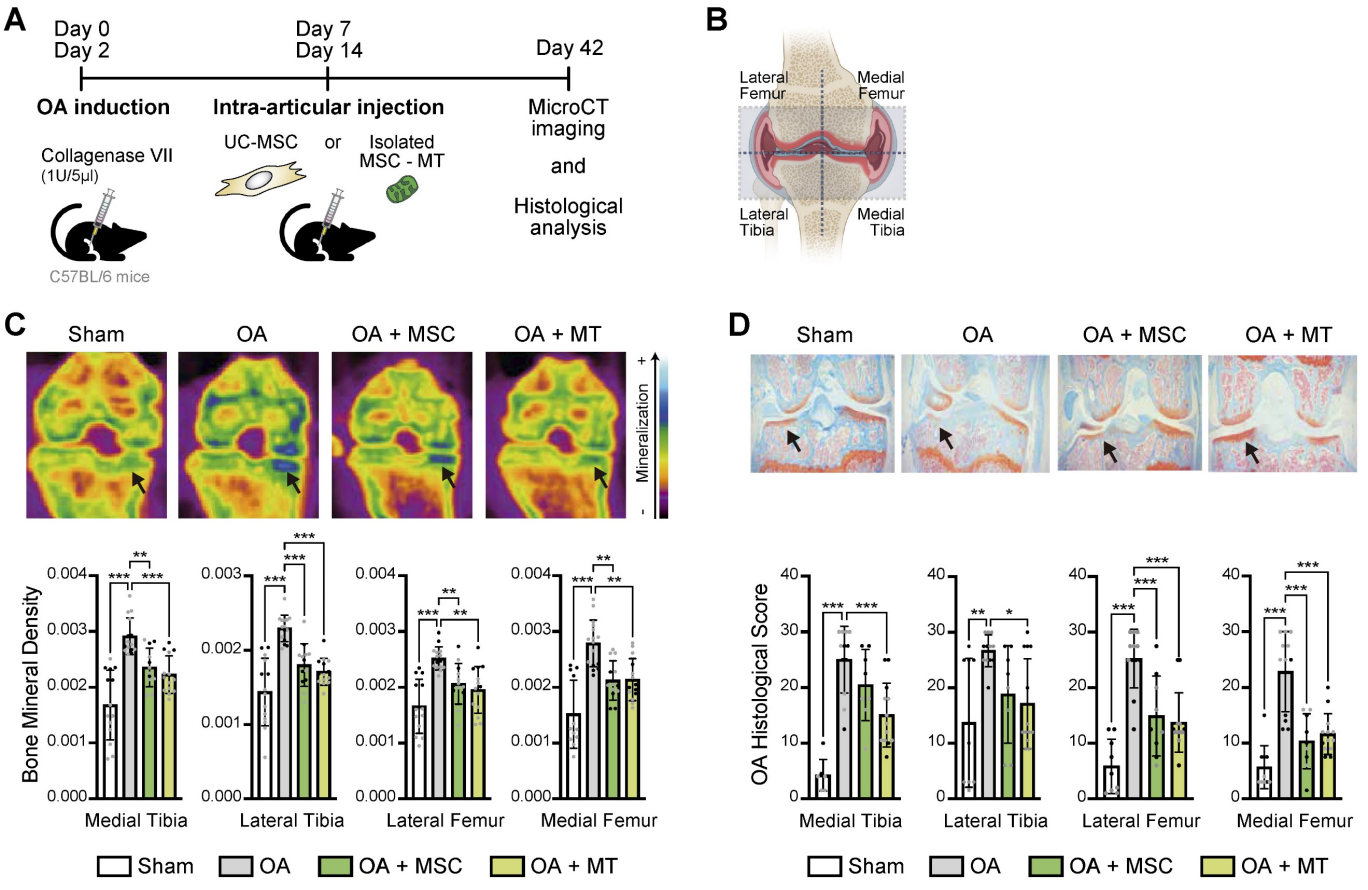
** Therapeutic effect of UC-MSC derived-MT treatment in a preclinical model of OA. (A)** *In vivo* experimental design of collagenase-induced model of OA (CIOA), treated with UC-MSCs or isolated human MSC derived mitochondria (MSC-MT). **(B)** Schematic representation of the articular joint zones subject to imaging and histopathological analysis. The femorotibial joint was divided into four zones: Medial Tibia, Lateral Tibia, Lateral Femur, and Medial Femur. **(C)** Bone mineral density analysis of CIOA mice (OA), CIOA mice transplanted intraarticularly with 2x10^5^ UC-MSCs (OA+MSC), CIOA mice transplanted intraarticularly with isolated MT derived from 2x10^5^ MSCs (OA+MT) or control group (sham, contralateral leg injected with sodium chloride). Representative 2D images of XY axes photography selection after MicroCT analysis (upper panel) and average of mineralization levels for each joint zone (bottom panel) (*n* = 2 experimental replicates, with at least 8 mice per group). **(D)** Representative histopathologic images (upper panel) and OA damage score quantification (bottom panel) in knee joint sections obtained from CIOA mice (OA), CIOA mice transplanted intraarticularly with 2x10^5^ UC-MSCs (OA+MSC), CIOA mice transplanted intraarticularly with isolated MT derived from 2x10^5^ MSCs (OA+MT) or control group (sham), for each joint zone (*n* = 2 experimental replicates, with at least 8 mice per group). Graphs show mean ± SEM and statistical analysis by non-parametric Mann-Whitney U test (*** p < 0.001; ** p < 0.01; * p < 0.05).

The significant effects on the OA-Ch triggered by MitoT led us to explore the translational potential of the intra-articular injection of UC-MSC-derived MT in a mouse model of OA. Confocal microscopy suggested the presence of labeled UC-MSCs-derived MT inside the chondrocytes harvested from MT-treated knee joints (Figures S4A-C), and qPCR confirm the detection of the human mitochondrial gene within the isolated murine Chs (Figures S4D-E). Functional MSC-derived MT were isolated and injected intra-articularly in the knee joints of mice with collagenase-induced knee OA (Figure 4A). We assessed the effect of MitoT on histomorphometric bone parameters by micro-computed tomography (MicroCT - μCT) and histopathological analysis. At day 42, both OA knee joints and sham non-treated knees were collected for imaging and histopathology (Figure 4A). We evaluated bone parameters in four sections of each knee, as observed in Figure 4B. While μCT images revealed the characteristic increase in knee bone mineralization in the OA mice groups, joints receiving MSC-derived MT injections (OA+MT group) exhibited a significant reduction (p < 0.01) in the bone mineral density (Figure 4C), that indicates less disease severity. Histological analysis of all knee sections also showed a significant decrease (p < 0.05) in the histological cartilage damage scores in the OA+MT group compared to the untreated OA mice (Figure 4D). In contrast, mice treated with UC-MSC (OA+MSC) did not achieve such inhibition of OA histological scores in medial and lateral tibia (Figure 4D).

Preliminary results showed that collagen of the cartilage matrix was increased in mice after 7 days post intra-articular injections of isolated MSC-MT (OA+MSC) compared to the untreated OA mice (Figures S5A-C). Furthermore, we observed that chondrocyte apoptosis induced in the OA knee joints tends to decrease after MT treatment (Figures S5D-E).

Altogether these results demonstrated the protective effect of MT transplantation on OA cartilage degradation, that was even stronger than the effect displayed by UC-MSC.

### MitoT grants oxidative stress resistance to OA chondrocytes, entailing anti-apoptotic effects

**Figure 5:**
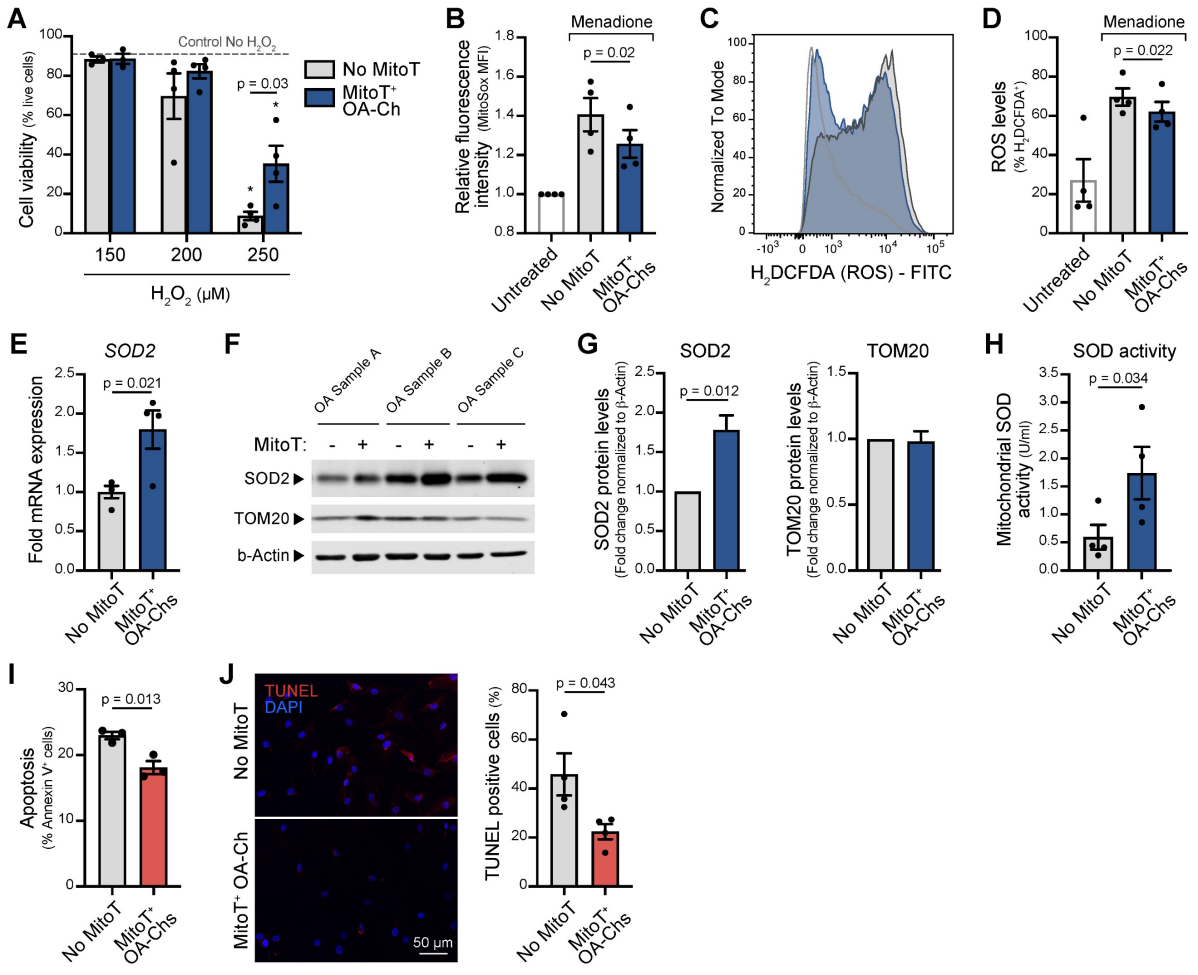
** MitoT increases resistance to oxidative stress in OA chondrocytes. (A)** Percentage of live cells on MitoT+ OA-Chs after 24 hours incubation with increasing concentrations of hydrogen peroxide (H_2_O_2_) compared to No MitoT control (*n* = 4 patient samples). * p < 0.001 compared to Control No H_2_O_2_ group. **(B)** Average mean fluorescence intensity (MFI) of MitoSox (2.5µM) by flow cytometry on MitoT+ OA-Chs and non-mitocepted Chs, treated for 30 minutes with menadione (MD, 25 µM), compared to untreated control (*n* = 4 patient samples). **(C)** Representative FACS histogram of ROS levels (measured with H_2_DCFDA) on MitoT+ OA-Chs compared to non-mitocepted Chs after incubation with MD, by flow cytometry analysis. **(D)** Average of the % H_2_DCFDA^+^ (ROS) population on MitoT+ OA-Chs compared to control. No MitoT after incubation with 25 µM MD, by flow cytometry analysis (*n* = 4 patient samples). **(E)** qPCR analysis of human superoxide dismutase 2 (SOD2) mRNA expression levels in MitoT+ OA-Chs after 48 hours post-mitoception with MSC-MT, compared to non-mitocepted control (No MitoT) (*n* = 4 OA patient samples). **(F)** Western blots of SOD2 and TOM20 mitochondrial proteins in MitoT+ OA-Chs after 48 hours post-mitoception with MSC-MT. b-Actin represented loading control. (*n* = 3 patient samples). **(G)** Fold change of SOD2 and TOM20 protein expression levels by Western blot analysis in MitoT+ OA-Chs after 48 hours post-mitoception with MSC-MT (*n* = 3 patient samples). **(H)** Mitochondrial superoxide dismutase activity levels (MnSOD) in MitoT+ OA-Chs and non-mitocepted Chs, after 24 hours post-mitoception with MSC-MT (*n* = 4 patient samples). **(I)** Percentage of apoptotic cells on MitoT+ OA-Chs after 3 hours incubation with 50 µM menadione compared to non-mitocepted control (*n* = 3 patient samples). **(J)** Analysis of apoptotic cell death by TUNEL assay of MitoT+ OA-Chs after 15 minutes incubation with 25 µM menadione, compared to No MitoT control. Representative confocal microscopy showed TUNEL-positive cells in red (left panel) and percentage of TUNEL-positive cells was calculated from four independent images per group (120-180 total cells analyzed) (right panel). Graphs show mean ± SEM and statistical analysis by Student's t-test. All replicates are biological.

Given the chondroprotective effect of the intra-articular injection of MT and that mitochondrial oxidative stress and increased apoptosis are a known common pathway of MT dysfunction in OA, we decided to assess the role of MitoT in the control of both phenomena (Figure S6A). To this end we exposed mitocepted OA-Chs to increasing concentrations of hydrogen peroxide (H_2_O_2_) (oxidative stress inducer) and observed a significant 4-fold increase in cell viability (p = 0.03) of MitoT^+^ OA-Chs compared to non-MitoT cells (Figure 5A and Figure S6B). Additionally, mitochondrial oxidative stress was significantly decreased (p = 0.02) in MitoT^+^ OA-Chs, when compared to non-MitoT cells treated with menadione (MD), a superoxide generating agent (Figure 5B and Figure S6C). Furthermore, reactive oxygen species (ROS) also decreased significantly (p = 0.039) in response to MitoT, as reflected in the shift of H2DCFDA fluorescence intensity in the FACS histogram analysis (Figure 5C and Figure S6D) and decreased ROS^+^ cell populations (p = 0.022) (Figure 5D and Figure S6E).

Since superoxide dismutase 2 (SOD2), a key suppressor of mitochondrial oxidative stress, is known to be decreased in the OA-Chs, we next tested the mRNA levels and protein expression of this antioxidant enzyme in response to MitoT. At 48 hours post-mitoception, qRT-PCR analysis revealed a significant upregulation (p < 0.05) of *SOD2* transcript levels in MitoT^+^ OA-Chs, compared to control non-MitoT OA-Chs (Figure 5E). The increase in SOD2 protein expression was consistently observed in three different primary OA-Chs culture samples (Figure 5F), with a 1.8-fold increase (p = 0.012) after MitoT (Figure 5G). Furthermore, using a colorimetric based SOD assay kit, we confirm that MitoT from UC-MSCs confers oxidative stress resistance to OA-Chs, since a significant 2.9-fold increase (p = 0.03) was detected for mitochondrial SOD activity levels (MnSOD) in MitoT^+^ OA-Chs (Figure 5H). The stable expression of another mitochondrial protein, TOM20 in control experiments confirmed that the increase in SOD2 was due to MitoT signaling and not merely to the uptake of more mitochondrial proteins derived from MSC-MT (Figure 5F-G). Finally, by annexin-based apoptosis assays and TUNEL staining, we evaluated if MitoT also conveys protection against cell death induced by oxidative stress in OA. As seen in Figure 5I, MitoT^+^ OA-Chs were significantly (p = 0.013) more resistant to menadione-induced apoptosis than non-MitoT cells. Confocal imaging of apoptotic cell death also confirmed the decrease (p = 0.043) in TUNEL-positive cells in OA-Chs after MSC-derived MitoT (Figure 5J). These results indicate that the control of oxidative stress induced by MitoT from UC-MSC confers protection against apoptosis in the OA-Ch *in vitro*.

## Discussion

Recent evidence points to decreased energy metabolism as the driving force of the catabolic imbalance of chondrocytes in OA 31. In a spontaneous knee OA model, chondrocyte ATP declines by approximately 50%, coupled with a reduced mitochondrial biomass per cell 7. Changes in the articular cartilage are believed to extend to subchondral bone and synovial tissue, creating a vicious circle of metabolic dysfunction, inflammatory changes and joint degradation. Not surprisingly, mitochondrial dysfunction underlies several pathways implicated in cartilage degradation 6,34, including mechanical stress, age and inflammation, leading to increased ROS and joint damage 2,6,15,34.

Recently, mitochondrial transfer and secreted small extracellular vesicles from MSCs to diseased tissues has been described as a mechanism involved in cellular rescue and tissue regeneration 35,36. In several models, MT transferred from MSCs promote a metabolic response of target cells by increasing basal respiration and ATP turnover 38. Wang *et al.* have recently demonstrated that bone marrow derived MSCs could transfer MT to rat OA-Chs in co-culture 39. While they claim that MT transfer is responsible for the beneficial effects on target OA-Chs, their experiments -in contrast with our data- only involve MSC co-culture that remains open to a host of MSC derived effects, not necessarily related to MT transfer. Previous research has provided *in vitro* evidence of the benefit of MitoT in mechanically damaged Chs 40. MitoT has been observed as a physiological response in the brain following stroke 41, while it has been recently shown that exogenous mitochondria traffic preferentially to sites that have sustained cell and tissue damage 42. Our data evidence a prompt (within 4 hours) cell-to-cell transfer of labeled MT from UC-MSC to human OA-Chs by flow cytometry and Confocal microscopy. Co-culture led to an increase in MT mass and copy number and a significant increase of ATP in diseased cells. This represents functional improvement in one of the hallmark traits of the OA-Chs, caused by a decrease in MT biomass and oxidative phosphorylation, despite the increase in glycolysis acting as a compensatory mechanism. The effects of MT transplantation seem to be dependent on their cell source 43 and UC-derived MSCs in particular exhibit higher mitochondrial ATP levels, basal and maximal respiration and mtDNA copy number 44.

To avoid artifacts due to dye leakage from donor cells, MitoT was also confirmed with MitoYFP-transfected donor UC-MSCs. These images evidenced tunneling nanotube (TNT-like) structures bridging donor MSCs and OA-Chs after co-culture. To assess the impact of MT on target cells without the interference of other cell-mediated but non-mitochondrial dependent actions, we separately probed chondrocyte function in response to artificial MitoT (mitoception). PCR assessment evidenced a significant increase of mitochondrial mass in target OA-Chs while Tetramethylrhodamine methyl ester (TMRM) MT stain for ΔΨm showed increased depolarization capacity of MitoT^+^ OA-Chs. This occurred in parallel with increased OXPHOS activity assessed by Seahorse technology, with no change in cell glycolytic capacity, pointing to a metabolic switch towards an activated energetic state. This suggests that the transfer of functionally active MSC derived MT can restore the metabolic dysfunction of the human OA-Ch. To address the fate of transferred MT within acceptor Chs we established a qPCR for donor specific single nucleotide polymorphisms (SNPs) enabling the detection of donor MT up to 9 days within target OA cells.

Even if increases in MT mass and MFI levels reached significance only at 24 hours of MitoT; exogenous MSC-MT were still detected until day nine when the OA-Chs where checked by SNP-PCR. This could be due to the heightened sensitivity of the SNP detection assay and its added capacity to discriminates exogenous from native MT withing the target cells. However, mitoception might also trigger intrinsic mechanisms by which the cell regulates MT-mass, such as mitophagy. Thus, the host cell could be eliminating the dysfunctional MT while retaining the transferred functional MT. Fate of MT is relevant, since Blanco *et al.* have shown that mitochondrial fission in the context of OA leads to mitophagy and energetic failure. In contrast, fusion improves mitochondrial function and energy production, inhibiting chondrocyte apoptosis 11. This lends significance to our finding of a predominantly fused state of the MT network in MitoT^+^ Chs by confocal microscopy, with concordant changes in the expression of MFN-2 and p-Drp1 proteins that control MT fusion and fission processes respectively. Fusion activity mediated by Mitofusin -while promoting an expanded MT network-, is known to enhance oxidative metabolism 45,46 and the synthesis of glutathione, a key antioxidant molecule, possibly restoring energy balance while inhibiting Ch apoptosis. Conversely, the dominance of mitochondrial fission is related to increased mitophagy in the context of OA, impairing calcium balance and redox state modulation 10,11, key components of the successful homeostatic response to stress 47. Furthermore, recent findings demonstrated that the mitochondrial fusion promotes chondrogenic differentiation of cartilage progenitor/stem cells and Mfn2 accelerates the chondrogenesis differentiation of these cell through Notch2. This was also confirmed *in vivo* by ameliorating OA in a rat model of the disease 48.

The wide range of restorative effects of MitoT on Ch function and dynamics prompted us to test the intra-articular injection of MT for a role in the treatment of a murine model of OA. Lee *et al.* have shown that intra-articular MT derived from OA patient's muscle tissue can reduce proinflammatory changes in a rat model of OA 49. However, no evidence of statistical changes in bone mineralization was observed. Furthermore, the authors did not demonstrate or show the presence of human MT in the joint tissues, although they attributed the histological improvement of the joints to the injected MT. There is also no indication of whether the MT are uptaken by chondrocytes or synoviocytes 49.

While most effects that we describe for MitoT seem mainly beneficial in OA, mitochondrial derived DNA can also exert a negative proinflammatory role in certain diseases 50. However very recently Fernández-Moreno *et al.* have shown that intra-articular injection of liver MT is safe in healthy mice 51. We have also shown previously 21 that MitoT activates T regulatory CD4^+^ T cells, while Westhaver *et al.* have evidenced that MT derived DNA, -a component of mitoDAMPs- promotes both natural killer and T regulatory phenotypes *in vitro* 52. These findings might be relevant to disease pathogenesis since intra-articular inflammatory phenomena are responsible not only for several disease manifestations but also for driving the pathophysiology of degenerative joint damage and disease progression 53. In this scenario, one of our main findings was the significant chondroprotective effect related to the intra-articular injection of isolated free MT. This also led to improvements in the reactive changes in bone mineral density that characterize disease in the collagenase-induced animal model of OA, as well as in human disease. Furthermore, we have been able to show first evidence that injected human MT are uptaken by the mouse chondrocytes *in vivo*. However, further tools need to be developed to show that those injected MT are metabolically active.

In human end-stage OA, chronic MT dysfunction has been associated with decreased SOD2: chondrocytes from patients undergoing hip replacement surgery exhibit SOD2 deficiency, which appears causally linked to oxidative damage and apoptosis mediated by MT-derived reactive oxygen species 34,54. Thus, we decided to assess the role of MitoT in the control of both phenomena in the OA-Chs, showing a robust effect of MT donation on the main constituents of this nociceptive pathway. MitoT was found to control mitochondrial oxidative stress, increasing SOD2 mRNA, activity and protein levels, finally reducing the levels of Ch apoptosis in response to stress inducing agents. These processes could be physiologically relevant since Fahey *et al.* have shown that bovine MSCs exposed to mechanically injured cartilage, localize to areas of matrix damage, delivering MT to Chs 40. In another study, the co-culture of stressed Chs with bone marrow-derived MSCs reversed MT failure. Improvement was attributed to the intercellular transfer of functional MT from MSCs to injured Chs and consequent recovery of ATP synthase and electron transport chain Complex I activity 55. Two limitation of our data are that in spite of blocking experiments with TNT inhibitors (cytochalasin) and connexin 43 blockers (carbenexolone, lantanum) aimed at the mechanism controlling MitoT, we were not able to entirely suppress the transfer of MT to OA-Chs. On the other hand, an aspect still under research is to what extent the effects described for MitoT^+^ Chs *in vitro*, are paralelled by the changes in the histopathology of diseased and treated OA mice *in vivo*.

While our work focuses on the tissue protective effect of MitoT through metabolic restoration, inflammation also presents an important facet of the disease. In patients with OA, targeting of cartilage and synovial cells, could modulate both the metabolic status of the Chs and the inflammatory cross-talk among joint compartments. In this regard, recent data from our group has shown that Mitoception with MSC-derived MT also triggers genetic reprogramming of target CD4^+^ T cells, leading to the development of functional T-regulatory (Treg) suppressor cells 21. While still speculative, such effect might contribute to dampen the ensuing cycle of synovial inflammation and cartilage degradation, described both in animal and human OA.

In summary, we describe for the first time, that Mitochondria isolated from clinically validated UC-MSCs 14,15 contribute to the control of chondrocyte homeostasis *in vitro* and in an animal model of OA. These effects are in line with the data that point to the effect of UC-MSC derived MT on restricting oxidative stress and its detrimental consequences on chondrocyte survival. Our findings are compatible with the notion that MitoT might play a physiologic role within the osteoarthritic joint, leading to new avenues of research while pointing to improved targeted interventions of the disease process in OA.

## Supplementary Material

Supplementary figures.
