# Supplementary material for: Mitochondrial transfer balances cell redox, energy and metabolic homeostasis in the osteoarthritic chondrocyte preserving cartilage integrity
Source: Theranostics. 2024 Oct 7;14(17):6471–86. doi: 10.7150/thno.96723 (PMC11519804; doi:10.7150/thno.96723)
Supplement: Supplementary file 1 — Supplementary figures. [file thnov14p6471s1.pdf]

Supp. Figure 1.

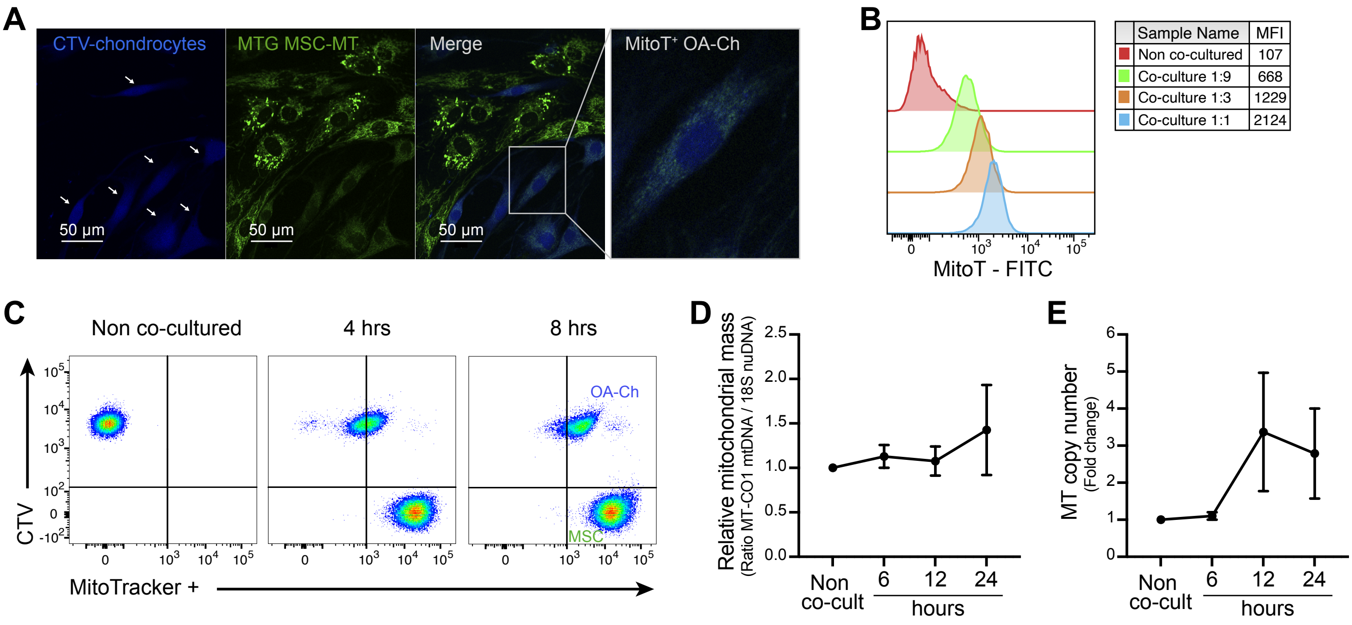

Supp. Figure 2.

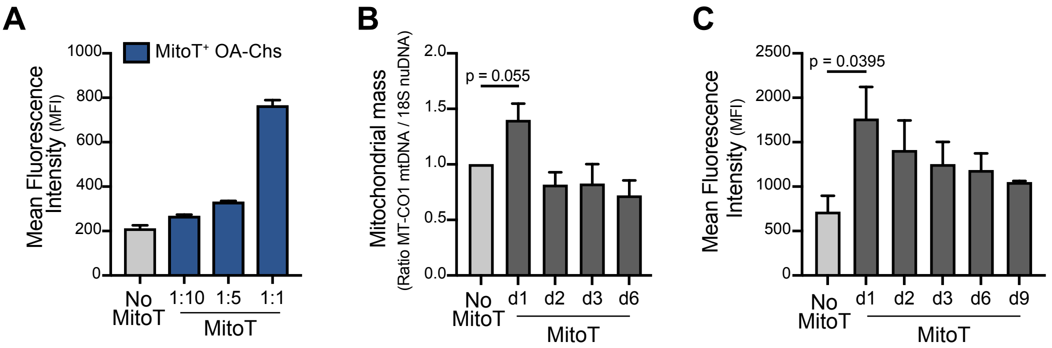

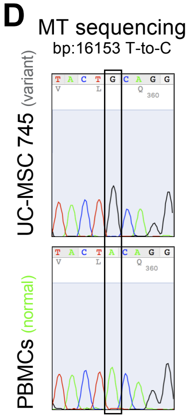

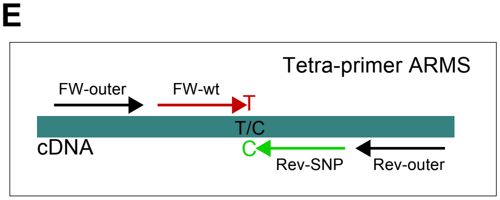

Transcript	Direction	Sequence (5´-3´)
16153 FW-outer	Forward	TAC ATT ACT GCC AGC CAC CAT GAA TAT T
16153 Rev-outer	Reverse	TAG TTG AGG GTT GAT TGC TGT ACT TGC T
16153 FW-wt	Forward	ACG GTA CCA TAA ATA CTT GAC CAC CGG T
16153 Rev-SNP	Reverse	TTT TGA TGT GGA TTG GGT TTT TAT GTA ATG
228 FW-outer	Forward	GTT CAA TAT TAC AGG CGA ACA TAC TTA CT
228 Rev-outer	Reverse	GTG GAA ATT TTT TGT TAT GAT GTC TGT
228 FW-SNP	Forward	AAA GTG TGT TAA TTA ATT AAT GCT TGG AA
228 Rev-wt	Reverse	CAG ACA TTC AAT TGT TAT TAT TAT GGC C

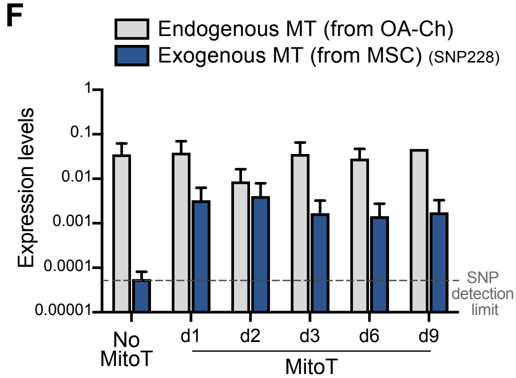

Supp. Figure 3.

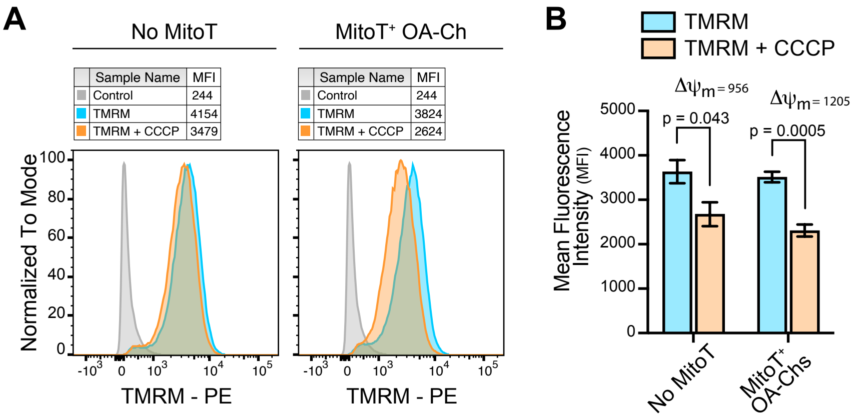

Supp. Figure 4.

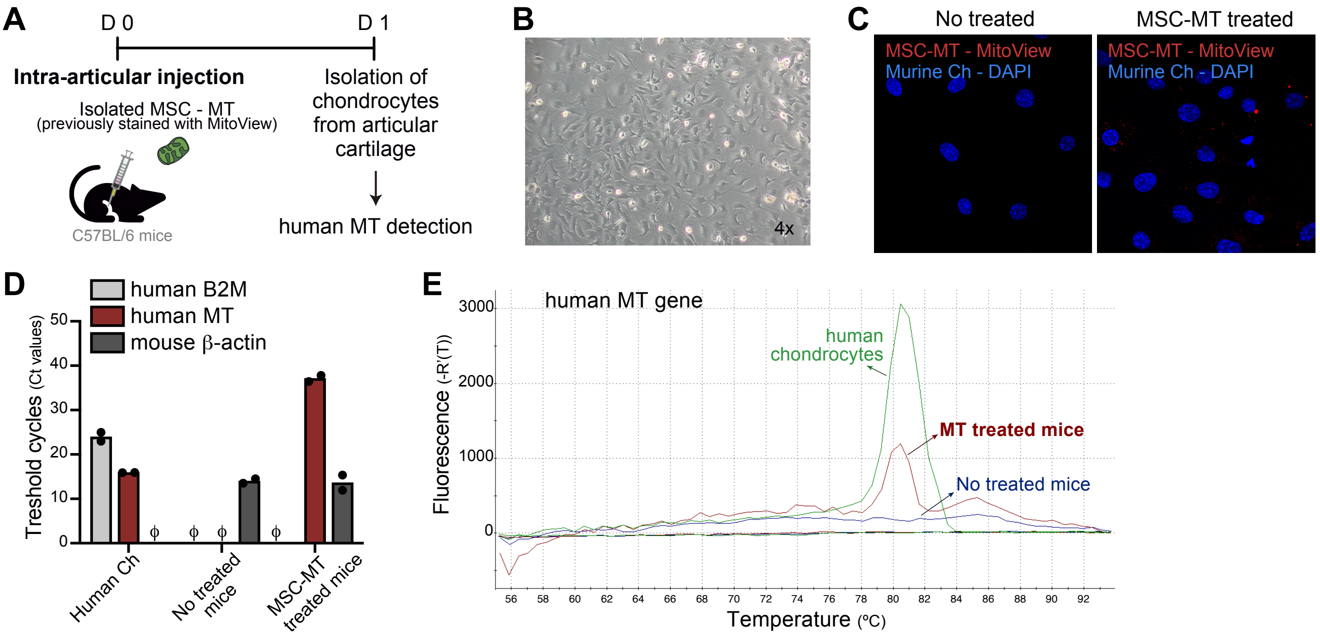

Supp. Figure 5.

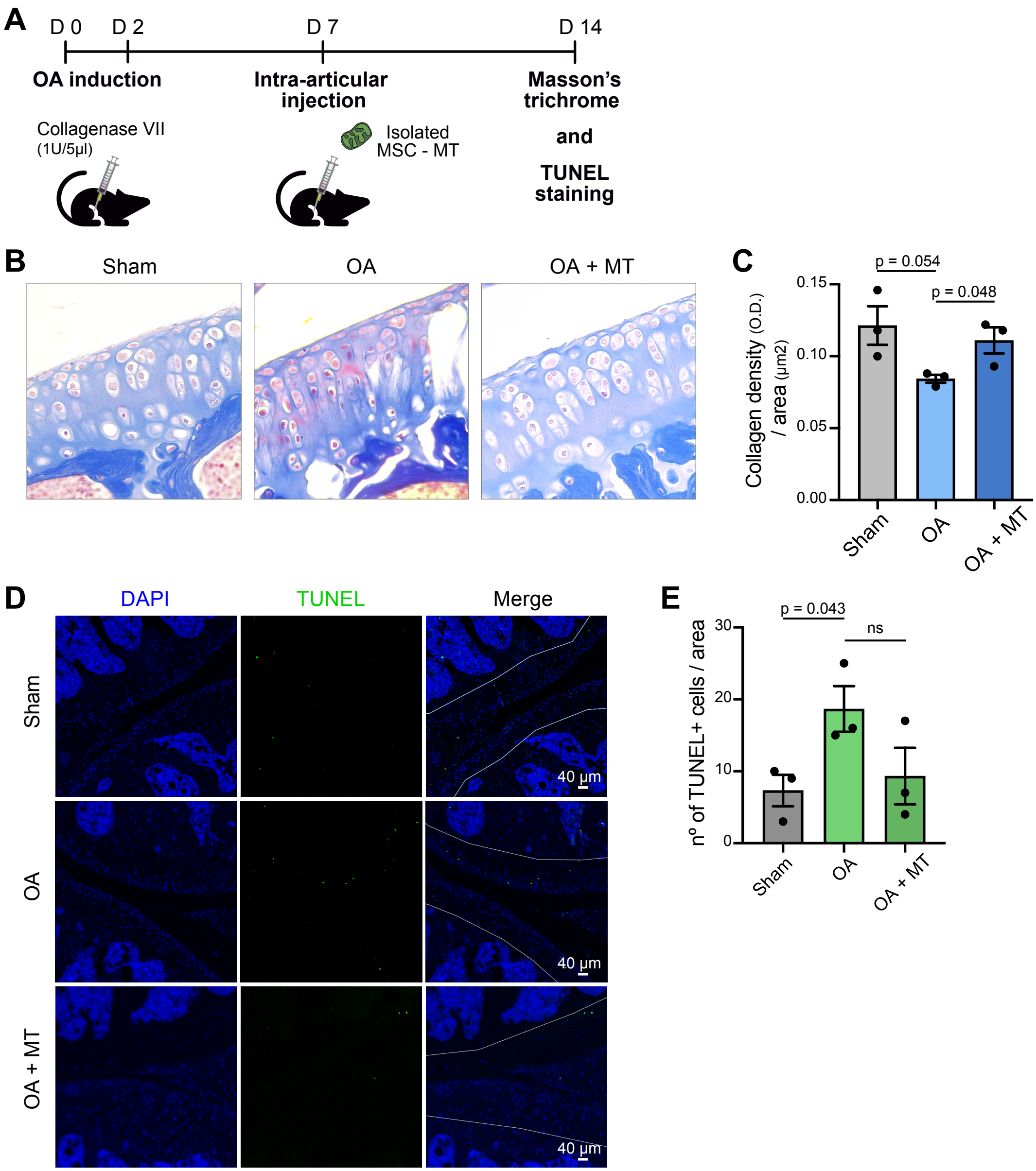

Supp. Figure 6.

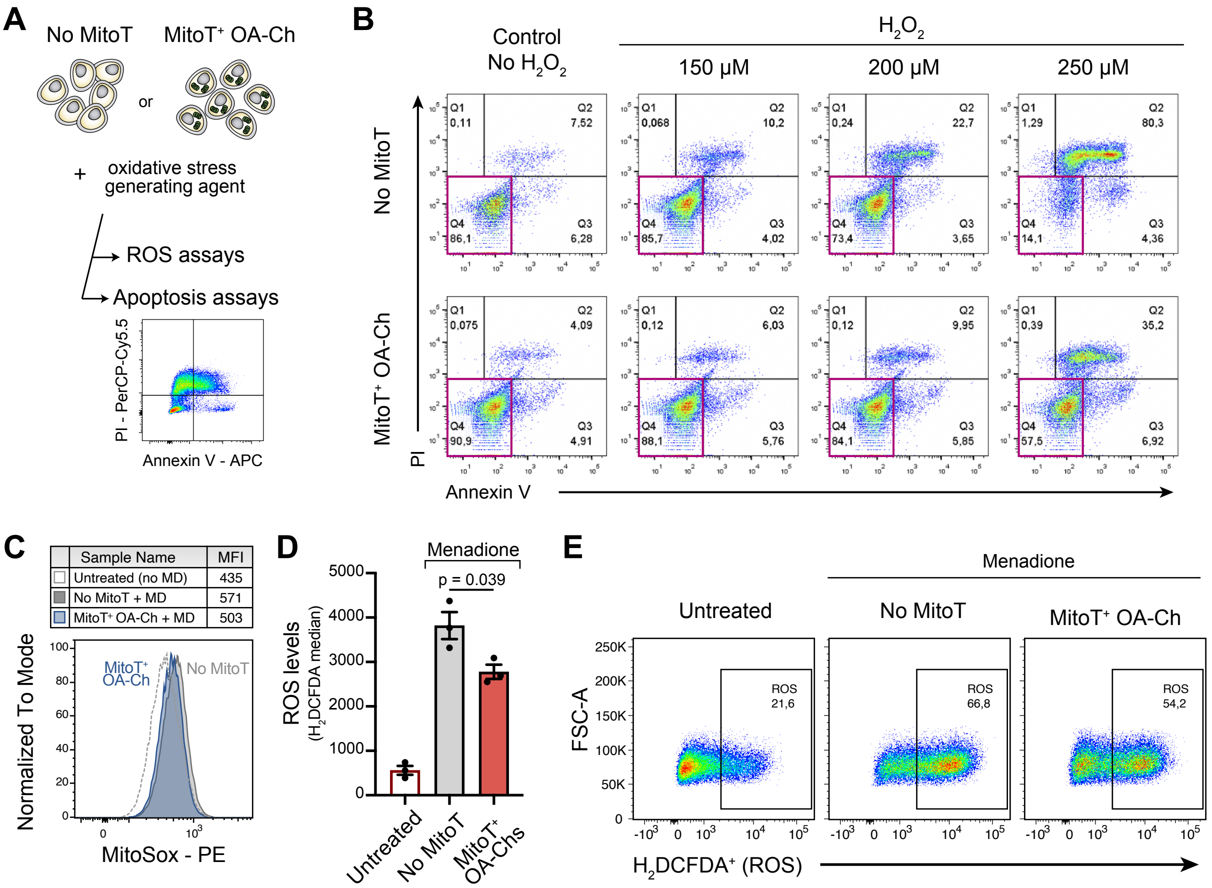

SUPPLEMENTARY FIGURE LEGENDS

Figure S1. MT Transfer from UC-MSC to human OA chondrocytes.

(A) Representative confocal microscopy of human CTV-stained OA-Chs (white arrows) co-cultured with UC-MSCs previously labeled with MitoTracker-Green (MTG) for 24 hours.
(B) Representative mean fluorescence intensity (MFI) of MitoT$^+$ OA-Chs at increasing co-culture ratios with MTG-labeled MSCs. Control-red histogram depicts OA-Chs with no MSC co-culture.
(C) Representative FACS plots of MitoT to CTV-stained OA-Chs at short co-culture times with MTG-labeled MSCs in a 1:1 ratio. Control OA-Chs with no MSC co-culture (left panel).
(D) Mitochondrial mass expressed as the mitochondrial DNA/nuclear DNA (mtDNA/nuDNA) ratio of MitoT$^+$ FACS-sorted OA-Chs at 6, 12 or 24 hrs of UC-MSC co-culture, as compared to control non co-cultured chondrocytes (n = 2 patient simples, replicates run in triplicates).
(E) Mitochondrial copy number of MitoT$^+$ FACS-sorted OA-Chs, at 6, 12 or 24 hrs of co-culture with UC-MSCs, compared to non co-cultured chondrocytes (n = 2 patient simples, replicates run in triplicates). Graphs show mean ± SEM and statistical analysis by Student's t-test. All replicates are biological.

Figure S2. Persistence of exogenous MSC-MT within OA-chondrocytes.

(A) Average MFI by FACS analysis of MitoT$^+$ OA-Chs transferred with increasing amounts of UC-MSC-derived MT, isolated from the equivalent number of MSCs according to previously tested cell ratios. Non mitocepted control in gray (n = 3 patient simples).
(B) Mitochondrial mass represented as the ratio of mitochondrial DNA/nuclear DNA (mtDNA/nuDNA) for MitoT$^+$ OA-Chs collected at different time-points (day 1-6) after mitoception with MSC-MT in doses equivalent to a 1:1 cell ratio (n = 3 patient simples).
(C) MFI by FACS analysis of MitoT$^+$ OA-Chs collected at days 1 through 9 after mitoception with MSC-MT in doses equivalent to a 1:1 cell ratio (n = 4 patient simples).
(D) Representative Sanger sequencing analysis of high-fidelity PCR products amplified with primers flanking the SNP 16153 T-to-C, showing G(C) peak in UC-MSC (code 745) cells, instead of an A(T) peak (in normal peripheral blood mononuclear control cells, PBMCs), allowing the identification of target acceptor cells versus donor MSC-MT.
(E) Primer design strategy (upper panel) and primer sequences (bottom panel) to selectively detect SNPs from MSC-MT gene from normal described MT sequence using the Tetra-primer Amplification Refractory Mutation System (ARMS) principles. FW = forward primer, Rev = reverse primer.
(F) Persistence of MSC-derived MT in OA-Chs according to SNP-PCR analysis of human-specific MSC mitochondrial-SNP (228 G-to-A) gene expression levels, in OA-Chs collected at different time points after mitoception, compared to non mitocepted chondrocytes (n = 3 patient simples). Graphs show mean ± SEM and statistical analysis by Student's t-test. All replicates are biological.

Figure S3. MitoT effects on human OA chondrocytes.
(A) Representative FACS histograms of the mitochondrial membrane potential, measured with Tetramethylrhodamine methyl ester (TMRM), on MitoT$^+$ OA-Chs at 24 hours post-mitoception with MSC derived MT compared to non-mitocepted OA-Chs control (No MitoT). MT membrane depolarization was induced with the mitochondrial oxidative phosphorylation uncoupler CCCP (100 μM).

(B) Average MFI of TMRM intensity levels for four different OA patients, as described above. DYm showed an increased depolarization capacity of MitoT^{+} OA-Chs compared to No MitoT OA-Chs control (*n* = 4 patient simples). All replicates are biological.

Figure S4. Human MT detection in mouse chondrocytes isolated from articular cartilage.
(A) *In vivo* experimental design of intra-articular injections with MSC-derived MT to evaluate its integration in the mouse joint.
(B) Bright-field microscopy image of primary cultured murine chondrocytes isolated from the knee articular cartilage (pooled samples from 3 mice), at day 6 after seeded for culture.
(C) Confocal microscopy images of chondrocytes isolated from MT-treated mice or control non treated mice, at day 6 after seeded for culture.
(D) qPCR analysis of human β2-microglobulin (B2M), human specific mitochondrial (MT) and mouse specific (β-actin) gene expression levels in chondrocytes isolated from MT-treated mice, no treated mice or human chondrocytes as control (*n* = 2). ϕ = not detected.
(E) Representative melting curves from qPCR of human MT gene from sample in (D).

Figure S5. UC-MSC derived-MT restores cartilage damage in a preclinical model of OA.
(A) *In vivo* experimental design of collagenase-induced model of OA (CIOA) treated with isolated human MSC derived mitochondria (MSC-MT), to evaluate cartilage damage by histological knee sections.
(B) Representative articular knee cartilage sections obtained from CIOA mice (OA), CIOA mice transplanted intraarticularly with isolated MT derived from $2x10^5$ MSCs (OA+MT) or control group (sham) stained with Masson's trichrome, showing positive stains for collagen (blue). (40x).
(C) Collagen density quantification calculated from two independent images per group described in (B). (*n* = 1 experimental replicate, with 3 mice per group).
(D) Representative confocal microscopy of TUNEL staining (green) in articular knee cartilage sections obtained from CIOA mice (OA), CIOA mice transplanted intraarticularly with isolated MT derived from $2x10^5$ MSCs (OA+MT) or control group (sham). Cell nucleus were stained with DAPI (blue). (10x).
(E) Quantification of the number of TUNEL-positive cells per unit area (500.000 µm) in the cartilage region (ROI) from the articular knee sections described in (D). (*n* = 1 experimental replicate, with 3 mice per group). In (C) and (E) graphs show mean ± SEM and statistical analysis by unpaired Student's t-test.

Figure S6. MitoT increases resistance to oxidative stress in OA chondrocytes.
(A) Experimental design of oxidative stress response in non-mitocepted OA-Chs (No MitoT) or MitoT^{+} OA-Chs.
(B) Representative FACS plots of apoptosis assay on MitoT^{+} OA-Chs after 24 hours incubation with increasing concentrations of hydrogen peroxide (H_2O_2) compared to No MitoT control.
(C) Representative histogram of MitoSox (2.5µM) by flow cytometry on MitoT^{+} OA-Chs and non-mitocepted Chs, treated for 30 minutes with menadione (MD, 25 µM), compared to untreated control.
(D) Average of the median fluorescence intensity of H_2DCFDA (ROS levels) on MitoT^{+} OA-Chs compared to non-mitocepted Chs after incubation with 25 µM MD, by flow cytometry analysis. (*n* = 3 OA patient samples). Graph shows mean ± SEM and statistical analysis by Student's t-test.
(E) Representative FACS plots of H_2DCFDA^{+} (ROS) population on MitoT^{+} OA-Chs compared to control No MitoT after incubation with 25 µM MD, by flow cytometry analysis.
